# A Computerized Frailty Assessment Tool at Points-of-Care: Development of a Standalone Electronic Comprehensive Geriatric Assessment/Frailty Index (eFI-CGA)

**DOI:** 10.3389/fpubh.2020.00089

**Published:** 2020-03-31

**Authors:** Katayoun Sepehri, McKenzie Sarah Braley, Betty Chinda, Macy Zou, Brandon Tang, Grace Park, Antonina Garm, Robert McDermid, Kenneth Rockwood, Xiaowei Song

**Affiliations:** ^1^Health Sciences and Innovation, Surrey Memorial Hospital, Surrey, BC, Canada; ^2^Department of Computing Science, Simon Fraser University, Burnaby, BC, Canada; ^3^Department of Biomedical Physiology and Kinesiology, Simon Fraser University, Burnaby, BC, Canada; ^4^Primary and Family Care, Fraser Health, Surrey, BC, Canada; ^5^Community Actions and Resources Empowering Seniors, Fraser Health Authority, Surrey, BC, Canada; ^6^Emergency Medicine, Surrey Memorial Hospital, Surrey, BC, Canada; ^7^Division of Geriatric Medicine, Dalhousie University, Halifax, BC, Canada; ^8^Centre for Healthcare of the Elderly, QEII Health Sciences Center, Halifax, NS, Canada

**Keywords:** aging, frailty, frailty index, comprehensive geriatric assessment (CGA), electronic assessment tools, healthcare, older adults

## Abstract

**Background:** Frailty is characterized by loss of biological reserves and is associated with an increased risk of adverse health outcomes. Frailty can be operationalized using a Frailty Index (FI) based on the accumulation of health deficits; items under health evaluation in the well-established Comprehensive Geriatric Assessment (CGA) have been used to generate an FI-CGA. Traditionally, constructing the FI-CGA has relied on paper-based recording and manual data processing. As this can be time-consuming and error-prone, it limits widespread uptake of this proven type of frailty assessment. Here, we report the development of an electronic tool, the eFI-CGA, for use on personal computers by frontline healthcare providers, to collect CGA data and automate FI-CFA calculation. The ultimate goal is to support early identification and management of frailty at points-of-care, and make uptake in Electronic Medical Records (EMR) feasible and transparent.

**Methods:** An electronic CGA (eCGA) form was implemented to operate on Microsoft's WinForms platform and coded using C# programming language. Users complete the eCGA form, from which items under the CGA evaluation are automatically retrieved and processed to output an eFI-CGA score. A user-friendly interface and secured data saving methods were implemented. The software was debugged and tested using systematically designed simulation data, addressing different logic, syntax, and application errors, and then tested with clinical assessment. The user manual and manual scoring were used as ground truth to compare eFI-CGA input and automated eFI score calculations. Frontline health-provider user feedback was incorporated to improve the end-user experience.

**Results:** The Standalone eFI-CGA software tool was developed and optimized for use on personal computers. The user interface adapted the design of paper-based CGA form to facilitate familiarity for clinical users. Compared to known scores, the software tool generated eFI-CGA scores with 100% accuracy to four decimal places. The eFI-CGA allowed secure data storage and retrieval of multiple types, including user input, completed eCGA form, coded items, and calculated eFI-CGA scores. It also permitted recording of actions requiring clinical follow-up, facilitating care planning. Application bugs were identified and resolved at various stages of the implementation, resulting in efficient system performance.

**Discussion:** Accurate, robust, and reliable computerized frailty assessments are needed to promote effective frailty assessment and management, as a key tool in health care systems facing up to frailty. Our research has enabled the delivery of the standalone eFI-CGA software technology to empower effective frailty assessment and management by various healthcare providers at points-of-care, facilitating integrated care of older adults.

## Introduction

It is estimated that the percentage of older adults over 65 years of age will double from 2016 to 2050 (1, 2). About a quarter of the older adult population is considered “frail” (3, 4), a state of diminished physiological reserve that impedes the body's ability to withstand or recover from acute stressors (5). Frailty can lead to various adverse health outcomes, including hospitalization and mortality (6, 7).

Several frailty-screening tools exist, including the Frailty Phenotype and the Clinical Frailty Scale (CFS) (8–12). The Frailty Index (FI) based on the accumulation of health deficits allows quantification of the degree of frailty; a person's level of frailty increases proportionally as their number of health deficits increases (13–15). The FI has been validated in studies across countries and healthcare settings (5, 16–21).

An FI can be calculated using items evaluated following a Comprehensive Geriatric Assessment (CGA) (22–24). The CGA evaluates multiple aspects of older adults' health (cognition, emotion, motivation, health attitude, vision, hearing, speech, sleep, pain, strength, balance, mobility, activities of daily living, social engagement, medication, control of life, etc.). The CGA allows a judgment-based assessment of a person's circumstances and leads to an understanding of the context in which potential impairments might arise. The CGA helps healthcare professionals develop an holistic overview of patients with complex needs, which is the essential step for the development of individualized, patient-centered care plans (25–28).

Construction of an FI using the CGA (FI-CGA) was developed for the Canadian Study of Health and Aging's clinical examination data (22, 23). The FI-CGA is highly correlated with a more generic FI (22–24), predicts length of stay and discharge destination in acute care settings, and has been validated using caregiver-completed health evaluations (29–34).

Despite its potential clinical utility, constructing the FI-CGA has traditionally relied on paper-based recording and manual data processing. As the process can be time-consuming and error-prone, it has presented a major deterrent for widespread adoption of the FI-CGA by the busy frontline clinicians.

In recent years, an increasingly significant attention has been paid to electronic frailty assessments. A team of United Kingdom researchers used electronic health records from millions of patients to execute an electronic FI (eFI) (35, 36). The eFI method was rapidly adapted by studies in other countries including the Scotland, Australian, and Canada, where large primary care datasets were extracted from Electronic Medical Records (EMR) and used to automate the eFI (37–39). More recent studies have tested the features of the eFI and validated the use of routine data in assessing frailty in aging and clinical practice research (40–44). The eFI undertaking can speed up frailty screening while revealing several consistent trends of the FI, such that patients with greater eFI scores were at a greater risk for mortality, hospitalization, and institutionalization (35, 36).

Even so, standard practice is to view the eFI as a screening tool that requires “eyes on” validation for decision-making at the individual patient level. This research indicates that developing an electronic FI-CGA (eFI-CGA) for *simultaneously evaluating the CGA and automating the FI* for clinical settings may allow a more feasible, time efficient, and cost-effective clinical assessment of frailty and benefit integrated health care of older patients.

We were therefore motivated to develop an electronic version of the CGA (eCGA) that can be use by frontline care providers to evaluate the CGA and automate an eFI-CGA output on personal computers, i.e., the Standalone eFI-CGA software tool. It was our intention to make eFI-CGA uptake in EMR feasible and transparent. Our specific objectives were to 1) design and implement an eCGA form based on a widely used traditional paper-based CGA form; 2) establish accuracy, robustness, and efficiency of the eFI-CGA calculation; and 3) optimize the eFI-CGA software tool for convenient use in clinical settings. This novel computer software tool is ready for use by frontline healthcare providers, to allow both computerized CGA evaluation and automated FI-CGA calculation.

## Methods

### Operating System, Platform, and Programming Language

As common practice in healthcare software application development, we first considered the operating system (OS), supporting software, and hardware available for the primary end-users (here, being healthcare professionals such as busy family physicians, geriatricians, and emergency doctors). Because the primary end-users will typically have access to a Microsoft Windows computer device, the Standalone eFI-CGA application was implemented to operate on Microsoft Windows 2000 or higher.

Given that many Microsoft Windows devices used in healthcare have enhanced security for installing additional applications due to privacy concerns, an ideal application needs to operate with the available Microsoft Windows libraries *per se*, rather than relying on additional third-party components. The two commonly used Windows .NET libraries for creating client applications are Windows Forms (WinForms) and Windows Presentation Foundation (WPF). Even though WPF would allow a great extent of flexibility in user interface design, the WPF applications rely on .NET framework 3.0 or later versions (https://docs.microsoft.com/en-us/dotnet/framework/winforms/), which may not be available on all devices running Windows 2000. Therefore, we chose to use WinForms, a platform upon which applications can be developed rapidly and executed with all computer devices supported by Microsoft Windows 2000 and .NET framework 2.0 or later versions (https://docs.microsoft.com/en-us/windows/desktop/choose-your-technology).

The eFI-CGA application was coded using C# programming language, a well-established, general-purposed, and object-oriented programming language for developing .NET applications. Microsoft's implementation of C# is integrated in the .NET framework, where the C# object model is consistent with the .NET object model. The available .NET abstraction allows for easy creation of complex objects (such as the selection button, dropdown list, etc) in the targeted software, so that Windows desktop applications can be developed conveniently using C# (https://docs.microsoft.com/en-us/dotnet/csharp).

### User Requirements Analysis

In developing the eFI-CGA application, we analyzed and satisfied several important user requirements, as detailed below.

#### General Appearance of the eCGA Form

**Figure 1:**
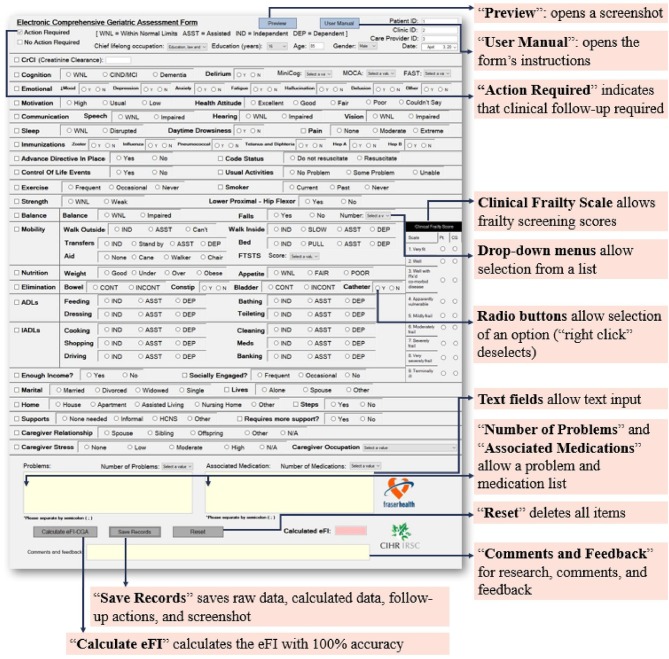
The main page of the eFI-CGA application. This interface presents a widely used Comprehensive Geriatric Assessment form (eCGA) and includes all the items under the CGA evaluation. It also includes the “Preview,” “User Manual,” “Reset,” “Save Records,” and “Calculate eFI-CGA” features, as highlighted. Also highlighted are the selection of critical eCGA items (e.g., the “Clinical Frailty Scale,” “Number of Problems,” and “Medications”), the flagging option for items that require follow-up an action in care plan, and the various input methods for the end-user.

First, the appearance of the electronic version must mirror a well-designed original paper-based CGA form familiar to clinicians. We chose to implement the Capital Health CD0184MR_06_09 form (Supplementary Materials #1), which is widely used, including in a randomized, controlled trial of CGA in rural community-dwelling older adults (45). A familiar user interface can help facilitate clinically meaningful evaluation and ease of completion, lessening the potential time, and computer skill constraints of the end-user. To satisfy this requirement, virtually each and every item in the electronic form was positioned in the same place as the template CGA form (Figure 1).

#### Control for User Input

Another key user requirement is enabling different field types for the form's various functions. Properly addressing this requirement can sufficiently realize the advantages of electronic methods over paper-based methods such that effective input-control is allowed. To satisfy the requirement, four major input methods were employed for specific fields, as appropriate (Figure 1). These included check boxes, radio buttons, text boxes, and drop-down lists. Check boxes were developed for item selections. Radio buttons promoting value selections were grouped using control containers, such that within each item, selection of only one of the grouped options at one time was permitted. Text boxes were given context-specific constraints as appropriate, e.g., only integers can be inputted into the “*Age”* field. Drop-down lists were developed to allow selection of an option from a short list of predetermined items. For example, when inputting cognitive assessment scores, the user can pick the correct value from the list of all possible values. The drop-down lists also allow for a categorization of inputs (e.g., occupation) to limit the range of responses and permit more straightforward data processing (Figure 1).

#### Clinical Follow-Up Actions

Having items needing clinical follow-up to be flagged is important for the end user to plan for care, helping direct the physician's attention to specific aspects of a patient that need management. To satisfy the requirement, additional check boxes with a blank note-space were created. Users can select multiple check boxes when multiple fields require follow-up and can also write more detailed records in the blank space (Figure 1).

### User Interface Design

**Figure 2:**
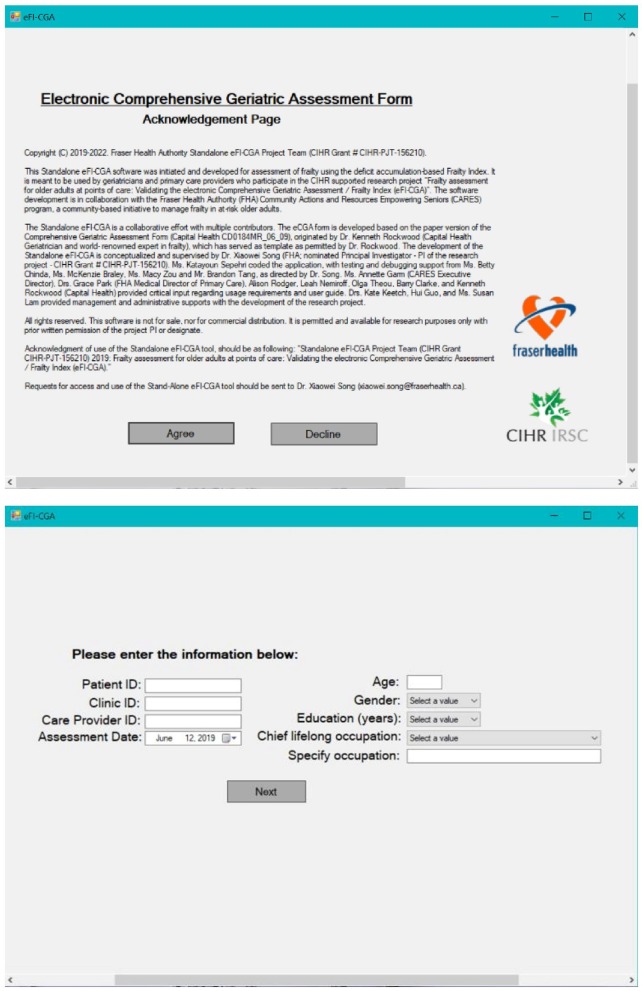
The other two pages of the eFI-CGA application. *The top panel* shows Page-1 of the application, which includes the statement of acknowledgments of use. *The bottom panel* shows Page-2 of the application, which is used to collect key background and demographic information needed for effective data management.

Aiming to achieve pronounced functionality and superb user experience with the software application, the graphical user interface (GUI) was built with three pages, each with a simple layout, consisting of a manageable number of actions, and targeting a specific function (Figures 1, 2).

#### Page 1

This page was developed to include the acknowledgments of use and to emphasize the non-commercial nature of the application (Figure 2). Choosing the “*Decline”* option closes the form. Only after agreeing to “*Accept”* the conditions of use, can the user continue the assessment.

#### Page 2

This relatively simple page was developed to gather the basic identifiable information about the patient (e.g., healthcare number, age, gender, education, etc.) and the clinician (e.g., clinician identification) necessary for data management (Figure 2). Only after all the fields on this page are filled out, can the user advance to the main page and access the eCGA form.

#### Page 3

This is the main page of the application that consists of the eCGA form and eFI-CGA calculator (Figure 1). The user can follow the page line-by-line to complete the form, and then press the “*Calculate eFI-CGA”* button at the bottom to view the eFI-CGA score. Additional features were also implemented on Page 3, such as “*Save Records”* which saves the input into various formats (see section Data storage for more details on data storage) and “*Reset”* which saves the inputs, clears the form, and sends the user to the previous page.

#### Pop-Up Pages

In addition, pop up features were made with Page 3. The “*Preview”* button opens one-page picture of the entire form in its current state, allowing the user to notice any trend in the data and to alert input errors. The “*User Manual”* button opens the user manual that describes eFI-CGA software usage and provides item-by-item instructions on completing the eCGA form.

### Dynamic User Guidance

**Figure 3:**
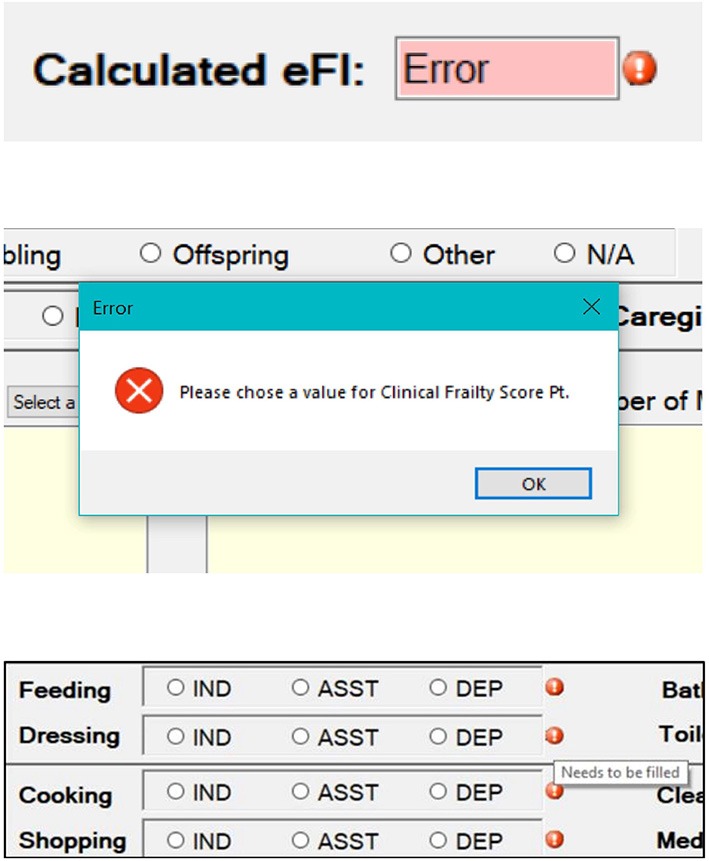
Images showing error- and warning-messages developed to guide the end-user to properly use of the eFI-CGA application. *The top section* shows an error message that appears when the user attempts to calculate the eFI-CGA without 80% of the form completed. *The middle section* shows a pop-up error message when the user is attempting to proceed without completing required fields. *The bottom section* shows symbols to remind the user of completing specific fields.

Multiple functions were developed to guide the user to complete the eCGA form dynamically. These functions include signifiers such as colored reminders, error messages, and warning signs. For example, an error message appears when the user attempts to proceed eFI calculation without finishing the assessment items (Figure 3), while colored reminders also appear next to the specific missing fields (refer to section Robustness handling for additional information on the error- and warning-messages).

### Calculation of the eFI-CGA

When implementing the eFI-CGA calculation the well-established deficit-accumulation-based FI approach was followed, as shown in the formula below: frailty is a proportion of the number of health deficits a person has out of the total number of health deficits under evaluation (12–16).

$$\begin{array}{l} \left[eFI-CGA\right]=\;\frac{\sum_{i=1}^{n}x_{i}}{n} \end{array}$$

Following the pre-determined FI-CGA variable coding scheme (Supplementary Materials #2), each raw input value was first mapped to a coded value between 0 and 1 (with values closer to one representing greater risk). The coded values were then stored in a list data structure. For example, coded values of “0,” “0.5,” and “1” were added to the list when the patient's “*Motivation”* was “*High,” “Usual,”* or “*Low,”* respectively. The question on the comorbidity count (i.e., “*Number of Problems”*) was also included as part of the deficits counts in the eFI-CGA calculation. To avoid a ceiling effect, the raw input value of comorbidity count was included in the deficit counts for the patient (unless it exceeded 18, in which case it was given a maximum assigned value of 18), while the maximum of 18 deficits was also included in the total number of deficits under evaluation (refer to Supplementary Materials #2). Here, assigning 18 as the maximum value is to balance the ratio of comorbidity diagnoses to the total deficits considered, here < $\frac{1}{4}$; it also represents a 99% limit to most contemporary methods of recording diagnoses. This ratio is typical with the community and clinical datasets studied previously, while in the CGA form comorbidities are recorded in a text field. The coded values and the count of comorbidities forming *n* deficits were used to generate the eFI-CGA (see formula above). The eFI-CGA score was calculated with precision to four decimal places.

Following the standard procedures for constructing an FI (46), an 80% non-missing value threshold guard was applied to the implementation. That is, the eFI-CGA was not calculated unless 80% of the form's required fields were completed. The system therefore checks if 80% of required fields is gathered before outputting a valid eFI-CGA. The system ignores missing fields when anywhere between 80 and 100% of the form is completed.

### Data Storage

For maintaining data security and confidentiality, the eFI-CGA application was not connected to the internet; instead, local data storage was implemented. Data are stored locally on the hard disk in comma separated value (CSV) format, which can be readily accessed with Microsoft Excel or a text editor available on all computers. Upon processing, all the raw data (the exact user inputs for each variable, allowing examination of possible input errors), calculated data (the coded values for debugging the eFI-CGA algorithm), action-required data (the items that require clinical follow-up), and a screenshot of the eCGA form (an image to allow recovery of user input and comparison of different assessments) are saved in separate files with time stamps. To minimize the possibility of data loss, the application also automatically saves the data once every 3 min and when the form closes or resets. Refer to section System input and output for more information on completion of data storage.

### Robustness Handling

To ensure that the software automates accurate eFI-CGA scores reliably and robustly, the Standalone eFI-CGA application was tested using a simulation and a clinical datasets, for which manual scoring following the coding scheme and the computing formula were used as ground truth to compare eFI-CGA input and automated eFI score calculations.

#### Testing Using Simulation Cases

The software tool was first tested using manually calculated test cases systematically created for system debugging purposes (*n* = 32). The data fall into four main categories and address specific conditions sensitive to system performance and accuracy, rather than real-world situations. The first category of test cases focused on debugging the 80% threshold guard of eFI-CGA algorithm and its ability to handle missing data. The category included cases where all the inputs were unanswered (i.e., 0% of the form completed) and where the number of completed inputs was at or barely below or above the 80% threshold. The second category addressed missing of entire sections of inputs, e.g., “*Mobility”* and “*Nutrition,”* to ensure that missing values representing a section would not affect the calculated eFI-CGA as long as the overall number of completed inputs was above the threshold guard. The third category examined consistency and flexibility of the software in handling different input values, including maximum and minimum input values allowed and other values within the range (i.e., 0, 0.5, 1 as with the coding scheme). The final category of test cases examined the response of the software in capturing and mapping correctly any raw input 1 to the coded value between 0 and 1 (Supplementary Materials #3). In addition, changes in the eFI-CGA in response to clinically meaningful input values were tested through manipulation of the valid values in a brute force manner.

#### Control for Usage Errors

In addition to ensuring that the software can output a correct score, efforts were made to minimize possible user errors in using the software tool that may affect eFI-CGA computation. To do so, logical input errors were prevented. For example, “*Number of Falls*” was automatically set to zero when the user inputs “*No*” for “*Falls*.” Also, although the threshold guard of eFI-CGA allows ≤20% missing input values, the form is responsive and guides the user with case-specific alert messages to prevent missing fields wherever possible; e.g., error- and warning-messages appear when the user attempts to proceed without making a choice, completing a field, or computing eFI-CGA score without 80% of the form completed (Figure 3).

#### End User Feedback

The application features, including methods regarding to the GUI, input and output, and data storage and handling, were optimized with clinical user feedback. The users, such as clinicians of primary, acute, and geriatric care settings, interacted with the application and provided valuable feedback addressing the application's simplicity and feasibility of use in clinical practice.

#### Debugging

When an unexpected output was observed or a revision was made for improved user experience, the code base of the involved application function and all the associated functions the of the program was checked, and the identified errors or changes were fixed. Then the test cases were applied to testing the software again, until accurate generation of all correct response was ensured.

#### Test in Clinics

To examine the usability of the software application in real-world situations, the software was made available in Fraser Health the Community Actions and Resources Empower Seniors (CARES) clinical intervention evaluation program (47). Preliminary data were collected in a small number of patient participants (*n* = 57; mean age = 79.4 ± 6.4 years, female = 45; mean education = 12.5 ± 3.3 years; with a Clinical Frailty Scale ranging between 3 and 6) by 14 clinicians at 6 clinics, with use of the delivered e-CGA tool. Outputs and time used were studied.

## Results

### System Input and Output

The Standalone eFI-CGA application was developed for use on personal computer devices. In all 32 cases, the 3-page GUI allowed user input of basic demographics of the patient, identification information for the healthcare professional, and all CGA items (Figures 1, 2). Several main features, including “*Preview,” “User Manual,” “Reset,” “Save Records,”* and “*Calculate eFI-CGA”* were enabled (Figure 1). Their implementations are summarized in Table 1.

**Table 1:** Implementation of the standalone eFI-CGA's main features.

**Main Feature**	**Purpose**	**Implementation**
Preview	Opens a one-page screenshot of the entire form for reviewing trends in the data and alerting to missing fields	When “Preview” is selected, the application saves a screen shot of the sections of the form and joins them together to create a single image.
User manual	Opens the user manual for item-by-item instructions on completing the eCGA items and usage of the Standalone eFI-CGA application	When “User Manual” is selected, the application opens the User Manual pdf file if it is pre-stored in the same folder of the software.
Calculate eFI-CGA	Automates an eFI-CGA score to 4 decimal places with 100% accuracy	When “Calculate eFI-CGA” is selected, the application maps inputs to coded values, uses the coded values in the FI-CGA algorithm, and outputs the eFI score.
Save records	Saves the inputs into files of various types	Once every 3 min, when “Save Records” is selected, and at the closure of the software interface, the application saves all inputs to various files.
Reset	Resets the application and returns the user to Page 2	When “Reset” button is pressed, the application clears all fields and opens Page 2.
Input validation	Prevents the user from entering incorrect information	The application ensures correct input format (e.g., string, integer) and the logical relationships between variables.
Missing data handling	Ensures that the user does not miss required information necessary for valid eFI-CGA calculation	When “Calculate eFI-CGA” is selected, the application checks every required field and prompts error messages if there are any missing fields.

**Figure 4:**
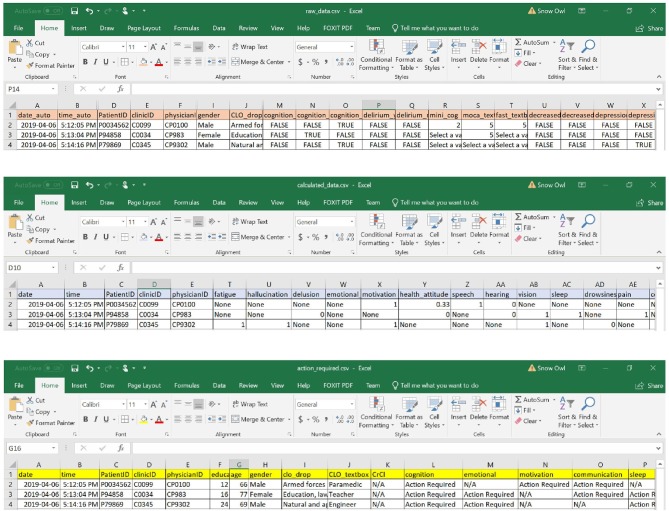
Examples of data files stored with the eFI-CGA application. *The top panel* displays the spreadsheet that contains the raw data of user input into the eCGA form throguh the applciation interface; e.g., with “TRUE” or “FALSE” as possible input value choices. *The middle panel* displays the spreadsheet that contains the automatically coded values for the input data; e.g., “1” or “0”; “none” indicates a missing value. *The bottom panel* displays the action-required spreadsheet that contains the items flagged as needing clinical follow up; e.g., “Action Required” or “N/A” indicating follow-up is required or not, respectively. Note that for privacy and confidentiality, the identifiers shown here are not real.

The inputs into the eCGA form were saved into four data files (Figure 4). The software tool saved raw inputs from the user (in the form of “*TRUE”* or “*FALSE”* for all possible input values), coded values (where “*none”* indicates a missing value), items that require clinical follow-up (where “*N/A”* indicates that no action was required), and a text summary list of all actions required. In addition, an image of the eCGA form in its final state was automatically saved upon closure of the application. In each spreadsheet, the date and time of the assessment were also successfully saved in the “*date”* and “*time”* columns to allow the user to revisit assessment items and compare scores (e.g., at various follow-up assessments).

**Figure 5:**
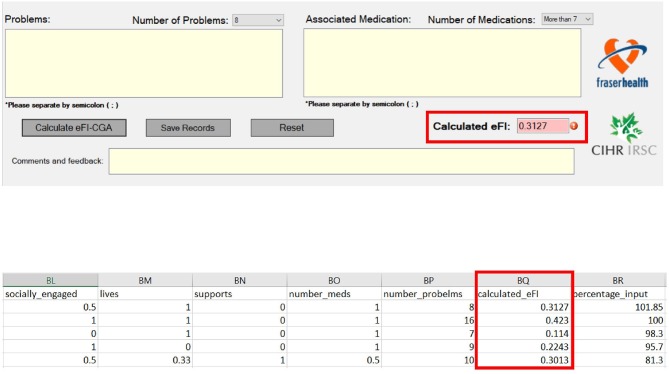
Frialty measurement scores as outputs of the eFI-CGA application. *The top panel* shows an image of the automated eFI-CGA score produced with a button-press by the user upon completion of inputing to the eCGA form. *The bottom panel* shows the saved eFI-CGA score with precision to four decimal places in the data spreadsheet.

After completing the CGA inputs, the software completed the eFI-CGA calculation and outputs the eFI-CGA score with precision to four decimal places, i.e., a number between the minimum possible value of 0.000 and the maximum possible value of 1.000 (Figure 5). As shown, the calculated score were displayed and simultaneously saved to both the raw data and calculated data spreadsheets (Figure 5).

### Performance

**Figure 6:**
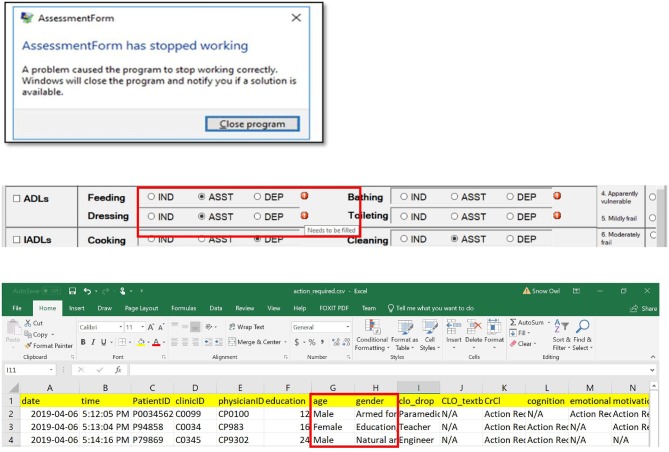
Common problems resolved during the implementation of the eFI-CGA application. *The top panel* shows an example of application crash detected and then resolved. When this occured, the user was forced to terminate the assessment and close the application. *The middle panel* shows an example of communication error that was detected and resolved, where a message erroneously popped up, notifying the user to complete fields that were already completed. *The bottom panel* shows an example of data misplacement that was corrected, where items were saved into a shifted column in the data file. Note that for privacy and confidentiality, the identifiers shown here are not real. Multiple application problems were identified and resolved owing to throughout software testing, so that the released software prevents all these errors from happening before delivered to the end-user.

Upon simulating each of the testing cases and interacting with the application, errors of six types were identified and resolved (Table 3). Typical errors included application crashing, functional errors, communication errors, score miscalculations, syntax errors, and incorrect data storage. With each error, an error message was shown, as demonstrated by examples (Figure 6). Types of errors, their causes and solutions, as well as the phase of their identification and correction during the software development are summarized in Table 2. Systematically identifying, diagnosing, and resolving the errors allowed the released version of the software to have 100% accuracy of the eFI-CGA calculation with precision to four decimal places applying all the test and clinical cases.

**Table 2:** Examples of system debugging and error handling.

**Category of the error**	**Example**			
	**Description**	**Cause**	**Resolution**	**Stage**
Application crashing	The application would crash when the user did not input a value into a drop-down down list.	Default value for dropdown lists was NULL.	The default value was changed from NULL to a string type “Select a value.”	Initial version
Unexpected function	When the user would close the form and then select “*No*” in response to the form's “*Do you wish to close the application?*” message, the form would move out of the window frame.	The form was attempting to save a picture when the user selected “*No”* to the close form confirmation message.	Disabled the image saving feature of the “*No”* response	Testing
Incorrect Communication	When the user responded to previously missing fields, error messages signifying missing fields would remain.	Spelling error in the code	Checked all error provider objects for correct variable assignments and ensured no interaction with other variables	Testing
eFI-CGA score miscalculations	The eFI-CGA score would not update after inputs were changed following original calculation.	Logic error in the code interpreting the formula	Checked variable mapping and ensured correct calculated value assignment	Testing
Syntax errors	“Creatinine” was spelled as “Creatine,” improper capitalization, etc.	Misspelling in the code of the GUI fields	Fixed the spelling error	Testing
Incorrect data storage	Data was saved under incorrect columns in spreadsheets	Wrong column ordering, or missing or added columns	Fixed the column ordering and added/removed columns	Testing

### Efficiency

Time complexity analysis revealed efficient performance with the Standalone eFI-CGA operations. Table 3 summarizes the time complexities for the eFI-CGA calculation, missing data handling, input validation, reset, and records saving operations, as detailed below.

**Table 3:** Efficiencies of the main features of the eFI-CGA application.

**Main features**	**Time complexity**
eFI-CGA calculation	*O(n)*
Missing data handling	*O(n)*
Input validation	*O(1)*
Reset functions	*O(m)*
Save records	*O(m + a + 2b)*

*Here “n” represents the total number of the possible value choices with all variable and text fields, “m” represents the number of all variables and text fields in the eCGA (where m < 215 in this application); “a” represents the number of variables in the eFI-CGA calculation (where a ≤ 60 in this application); and “b” represents the number of required actions (b ≤ 35 in this application)*.

Transformation of the raw input values into a coded value between 0 and 1 requires multiple values for each variable to be visited once and stored in an array with a time complexity of *O*(*n*), were “n” represents the total number of the possible value choices with all variable and text fields. Similarly, summing the coded values of *m* variable and text fields stored in the array based on the FI formula had a complexity of *O*(*m*), were “m” represents the number of the variable and text fields. In addition, by pressing the “*Calculate eFI-CGA”* button, the application checks for missing fields and generates specific error messages, so that the variable is revisited. In total, the time complexity for “Calculate eFI-CGA” is limited to *O*(*n* ).

To verify the validity of the user input values to the eCGA form, the application checks whether each input is logically meaningful and appropriately formatted. This operation is done in constant time *O*(1), through simple comparisons and matching between the input and the pre-defined requirement (Supplementary Materials #2).

The “Reset” operation had *O*(*m*) time complexity as all input fields must been visited once when their values were cleared.

The “Save Records” operation is also efficient (Table 3) with a time complexity of *O*(*m* + *a* + 2*b*), as it produces saving in different files with different formats into multiple spreadsheet and test files (where *a* and *b*, respectively, represents the number of variables using in frailty calculation and the total number of action items). While all the variables in the eCGA form were saved once (i.e., raw data), as were the variables used in the eFI-CGA calculation (i.e., coded data), the items requiring follow-up actions were saved twice in separate and text files (i.e., original input and action needed files).

### Clinical Test

Of the 57 patients tested so far, the automated eFI-CGA data matched the known ground truth FI to the fourth decimal point (Table 4), demonstrating a 100% accuracy rate. The patients showed a mean eFI-CGA score of 0.2516 ± 0.1123. There were on average 7.9 ± 3.7 comorbidities with these patients; 0.73 ± 1.36 action fields were identified for clinical follow-up. The average time spent automating an eFI-CGA score through completing an eCGA assessment was 44.5 ± 12.8 min, whereas the time spent to automate the eFI-CGA score by entering pre-recorded CGA items was 8.6 ± 3.3 min, demonstrating feasible use of the software tool in clinical applications.

**Table 4:** Initial tests of the standalone eFI-CGA software application in medical clinics.

**Patients (*n* = 57) 45 females; 12 males**	**Mean**	**S.D**.	**Minimum**	**Maximum**
Age (year)	79.4	6.4	65	97
Education (year)	12.5	3.3	4	22
Clinical frailty scale	3.69	0.87	3	6
Comorbidities	7.9	3.7	1	18
Number of actions needed	0.73	1.36	0	6
eFI-CGA	0.2516	0.1123	0.0429	0.5783
Time for assessment (minute)	44.5	12.8	20	66
Time from item entry (minute)	8.6	3.3	5	24

## Discussion

In this study, we developed an eFI-CGA software application to assist frontline frailty assessment and care planning. The eFI-CGA software tool takes user input values from the electronic form of CGA interface and automates the data recording, storage, process, and calculation. The software outputted an reliable eFI score, together with several files including an image of the complete eCGA form and areas to follow-up with care plan, The application was implemented on the Microsoft's WinForms platform for convenient operations on personal computers. For user friendliness and familiarity, the graphical user interface of the software represented the design of an established paper-based CGA form. Testing and debugging with the application of systemically designed testing data, real-world data, initial clinical data, and user feedback revealed that the eFI-CGA calculation to have 100% accuracy with precision to four decimals. The Standalone eFI-CGA software application allowed the user input to the eCGA form to be saved multiple times at various data inputting and processing stages, benefiting future reference and check. Following clinician feedback, the eFI-CGA software delivery was considered to be efficient and suitable for use in clinical settings.

Frailty is a significant challenge for successful healthcare of older adults with complex health conditions and care needs. In spite of studies showing that frailty can be improved when identified and managed at an early stage (13), there is presently a lack of clinically feasible and valid frailty assessment tools to facilitate care planning. Currently, electronic versions of frailty measurement that yield an FI score are based on *ad hoc* assessment, not a CGA that has been demonstrated to be effective in trials. Recent research using existing electronic health records to execute eFI has speeded up frailty screening (35–44). Current effort is focused on validation for decision-making at the individual patient level. Conducting CGA assessments is the main intervention in geriatric medicine, where it is still often done using paper forms, or with scribes. Our technological paper benefits from a strong understanding developed with frontline users, one of who developed the original paper version in the 1990s (45).

The FI-CGA is nowhere part of standard primary care; the UK eFI (35), for example, although developed from general physician records, is seen as a screening tool that requires verification of people who screen positive with an assessment. Our motivation here is part of a wider effort to “geriatrize” routine care, with a goal to move early management of frailty toward an enhanced primary care. To do so will require appropriate tools. The software tool development therefore aims to transition what has worked on paper, in the hands of geriatricians, to the EMR as an eFI-CGA. Our research has enabled the delivery of the eCGA and the eFI-CGA software, as automated geriatric and frailty measurement tools, to empower effective management of frailty at points-of-care. Even after EMR version is made available, its use by physicians of primary care and acute care helps integrate information across healthcare settings that can inform comprehensive, individualized care plans.

The Standalone eFI-CGA software application addresses a few urgent needs in frailty research. It can serve as a platform to compare outputs of different eFI-CGA implementations employed by multiple EMR vendors without a standard, and with the usual function / system trade-offs that are endemic to implementation in that context. Currently, vendors of different EMR systems are automating the eFI-CGA on EMR follow different approaches that can be unwieldy, making it difficult to merge the measurements outputs. Future analyses will compare different EMR-embedded with the Standalone eFI-CGA versions, to ensure feasibility and transparency of the implementation and accurate and robust in outputting eFI-CGA scores. In addition, the eFI-CGA software tool also allows more effective data collection with eCGA and eFI-CGA. For example, healthcare professionals can start data collection as long as they have a Windows-based desktop or laptop computer to operate with, in any appropriate environment, e.g., even patient homes, without being limited by an access difficulty, e.g., to a particular EMR.

Employing the eFI-CGA in clinical programs can benefit effective assessment of intervention results. In the Community Actions and Resources Empower Seniors (CARES) clinical intervention evaluation program (47), healthcare professionals develop individualized treatment plans and make referrals to community-based programs based on eFI-CGA results (48, 49). The eFI-CGA is calculated at various points in time to monitor changes in frailty as an outcome of the CARES intervention. Once validated for widespread clinical use, the eFI-CGA may become a part of standard practice for integrated healthcare of older adults including primary care and home medicine, to promote early identification of frailty, which would subsequently improve informed decision-making and care planning. More specifically, the eCGA form that can automatically output an FI-CGA measurement, allowing healthcare professionals to tailor interventions to their patients' individual needs. As the Standalone eFI-CGA software tool can be conveniently executed on any personal computer, it can be readily adopted by a range of healthcare professionals in different settings.

Several technical limitations with software application should be noted and warrant further development and implementation effort. First, the efficiency of the application could be further improved in future releases with smarter implementation techniques. For example, “*Calculate eFI-CGA*” could check for missing inputs while mapping each variable in the same step. Even so, as the number of variables in the eCGA form is small, the improved efficiency of the calculation may not have an impact on the user experience. The eFI-CGA score is produced quickly on almost all devices within milliseconds of pressing the calculate button in its present form. The time complexity for other features, such as saving records or resetting, is also efficiently *O(n)* and equally not likely to show enhancements with improved implementations.

Currently, simultaneous reading and writing to the same memory location is prohibited by the operating systems, and thus attempting so prompts an error message by the software program. That is, one cannot open the saved data files at the same time when the software is being used, due to its automatic background safeguard process that periodically saves data to these files (once every 3 min). To prevent such potential problem, the data files are made hidden from the user at the time of value input; they become available once data entry is complete and program is closed). A cleaner approach for future implementation could be to store the data separately on a server or in the AppData folder, which is hidden by default on all computer devices and contains application settings, files and data. However, such an operation would require the program to access and run from the host computer, not just a memory stick attached to the computer as presented with the current design.

Using WinForms as the main platform to develop this application also involved inherited challenges in the GUI design. The eCGA form may appear marginally differently or slightly blurry depending on the resolution settings of the user's device. Furthermore, some features could be enriched to improve the user experience, such as a zooming in/out function, and may be more feasible to implement with newer version of WinForms. As healthcare technology advances and later versions of Microsoft Windows become accessible to all users, platforms such as WPF libraries will allow more flexibility in GUI design.

Taken together, importantly for future development of research line, before the eFI-CGA can be implemented into standard healthcare practice, the psychometric properties must be established. With the use of a standalone software tool that can be used with any personal computer, the eFI-CGA scores can be tested in healthcare settings by different healthcare professionals, making it useful for reliability and validity analyses. Our ongoing research is to complete the collection of longitudinal clinical assessment data to examine the reliability and validity of the eFI-CGA tool and its clinical applications. In related work, we are building and testing the eFI-CGA on EMR with transparency and feasibility, taking insights of the present software development.

### Restrictions for Use

The Standalone eFI-CGA application was initiated and developed as part of the research project “Frailty assessment for older adults at point of care: Validating the electronic Comprehensive Geriatric Assessment / Frailty Index (eFI-CGA).” The software development was in collaboration with the Fraser Health Authority (FHA) Community Actions and Resources Empowering Seniors (CARES) program, a community-based initiative to manage frailty in at-risk older adults and Nova Scotia Health Authority (NSHA) Geriatric Medicine Research Unit (GMRU). All rights are reserved to the Stand-Alone eFI-CGA Project Team (CIHR Grant # CIHR-PJT-156210 2018-2022). The Standalone eFI-CGA software is not for sale, nor for commercial distribution. The released software product is available for research, academic, and clinical purposes with prior written permission of the project's primary investigator (XS, the corresponding author) or designate. Parties interested in using the software tool should contact the primary investigator (xiaowei.song@fraserhealth.ca) to receive a Standalone eFI-CGA Research Agreement Form. After signing the research agreement, the party will receive a link to the encrypted application. Use of the eCGA / eFI-CGA software in publications and media communications should state the following: “The eFI-CGA Project Team (CIHR Grant CIHR-PJT-156210) 2018-2022: Frailty assessment for older adults at points-of-care: Validating the electronic Comprehensive Geriatric Assessment / Frailty Index (eFI-CGA).”

Readers are welcome to access the software for research purposes, by following the link below.

https://docs.google.com/forms/d/e/1FAIpQLSf4KLsFNd8-z4xnYvbyB1JZITUaVWZNJQnk-sDq82INDJlNgQ/viewform?usp=sf_link

Please email the correspondence author: xiaowei.song@fraserhealth.ca, to obtain the password to download the software from the link above.

## Data Availability Statement

All datasets generated for this study are included in the article/Supplementary Material.

## Ethics Statement

This study was approved by Fraser Health Human Research Ethics Board (FHREB2018-080). The patient and clinician participants provided their written informed consent to participate in this study.

## Author Contributions

KS coded the application and drafted the technical aspects of the manuscript. MB, BC, MZ, and BT provided testing, debugging, data processing and analysis support, and helped prepare the manuscript. GP, AG, and RM provided critical input regarding user requirements, user manual and clinical data collection, and edited different versions of the manuscript. KR originated the paper-based CGA form and allowed the research team to use the form as a template for developing the software, provided critical input regarding user requirements and guidance, clinical data collection, and edited the manuscript. XS conceptualized and supervised the development of the eFI-CGA software application, obtained research funding, supervised data processing, analysis, and co-prepared the first draft of the manuscript. All authors reviewed and approved the submission of the final version of the paper.

### Conflict of Interest

The authors declare that the research was conducted in the absence of any commercial or financial relationships that could be construed as a potential conflict of interest.
