# Supplementary material for: A Computerized Frailty Assessment Tool at Points-of-Care: Development of a Standalone Electronic Comprehensive Geriatric Assessment/Frailty Index (eFI-CGA)
Source: Front Public Health. 2020 Mar 31;8:89. doi: 10.3389/fpubh.2020.00089 (PMC7137764; doi:10.3389/fpubh.2020.00089)
Supplement: Supplementary file 1 [file Table_1.pdf]

Supplementary Materials #2, Table 1_v1 (The eFI-CGA Variable Coding Scheme)

Variable Name	Field Type	Data Class	Raw Input Values	Coded Values
Cognition	Radio button	Descriptive	Within Normal Limits	0
			CIND / MCI	0.5
			Dementia	1
Delirium	Radio button	Descriptive	No	0
			Yes	1
Mini-Cog	Dropdown list	Numerical [0, 5]	5	0
			4	0.33
			2-3	0.66
			≤1	1
MOCA	Dropdown list	Numerical [0, 30]	≥25	0
			20-24	0.33
			11-19	0.66
			≤10	1
FAST	Dropdown list	Numerical [1, 7]	1-2	0
			3-4	0.5
			≥5	1
Low Mood	Radio button	Descriptive	No	0
			Yes	1
Depression	Radio button	Descriptive	No	0
			Yes	1
Anxiety	Radio button	Descriptive	No	0
			Yes	1
Fatigue	Radio button	Descriptive	No	0
			Yes	1
Hallucination	Radio button	Descriptive	No	0
			Yes	1
Delusion	Radio button	Descriptive	No	0
			Yes	1
Other	Radio button	Descriptive	No	0
			Yes	1
Motivation	Radio button	Descriptive	High	0
			Usual	0.5
			Low	1
Health Attitude	Radio button	Descriptive	Excellent	0
			Good	0.33
			Fair	0.66
			Poor	1
Speech	Radio button	Descriptive	Within Normal Limits	0
			Impaired	1

Hearing	Radio button	Descriptive	Within Normal Limits	0
			Impaired	1
Vision	Radio button	Descriptive	Within Normal Limits	0
			Impaired	1
Sleep	Radio button	Descriptive	Within Normal Limits	0
			Disrupted	1
Daytime Drowsiness	Radio button	Descriptive	Yes	1
			No	0
Pain	Radio button	Descriptive	None	0
			Moderate	0.5
			Extreme	1
Control of Life Events	Radio button	Descriptive	Yes	0
			No	1
Usual Activities	Radio button	Descriptive	No Problem	0
			Some Problem	0.5
			Unable	1
Exercise	Radio button	Descriptive	Frequent	0
			Occasional	0.5
			Not	1
Smoke	Radio button	Descriptive	Never	0
			Past	0.5
			Current	1
Strength	Radio button	Descriptive	Within Normal Limits	0
			Weak	1
Lower Proximal – Hip Flexor	Radio button	Descriptive	No	0
			Yes	1
Balance	Radio button	Descriptive	Within Normal Limit	0
			Impaired	1
Falls	Radio button	Descriptive	No	0
			Yes	1
Falls Number	Dropdown list	Numerical [0, 99]	0	0
			1	0.5
			>1	1
Walk Outside	Radio button	Descriptive	Individual	0
			Assistance	0.5
			Can’t	1

Walking Inside	Radio button	Descriptive	Individual	0
			Slow	0.33
			Assistance	0.66
			Dependent	1
Transfers	Radio button	Descriptive	Individual	0
			Stand by	0.33
			Assistance	0.66
			Dependent	1
Bed	Radio button	Descriptive	Individual	0
			Pull	0.33
			Assistance	0.66
			Dependent	1
Aid	Radio button	Descriptive	None	0
			Cane	0.33
			Walker	0.66
			Chair	1
FTSTS	Dropdown List	Numerical [0, 15]	≤9	0
			10-14	0.5
			≥15	1
Weight	Radio button	Descriptive	Good	0
			Under	0.5
			Over	0.5
			Obese	1
Appetite	Radio button	Descriptive	Within Normal Limit	0
			Fair	0.5
			Poor	1
Bowel	Radio button	Descriptive	Continent	0
			Incontinent	1
Constipation	Radio button	Descriptive	No	0
			Yes	1
Bladder	Radio button	Descriptive	Continent	0
			Incontinent	1
Catheter	Radio button	Descriptive	No	0
			Yes	1
Feeding	Radio button	Descriptive	Individual	0
			Assistance	0.5
			Dependent	1
Bathing	Radio button	Descriptive	Individual	0
			Assistance	0.5
			Dependent	1
Dressing	Radio button	Descriptive	Individual	0
			Assistance	0.5
			Dependent	1

Toileting	Radio button	Descriptive	Individual	0
			Assistance	0.5
			Dependent	1
Cooking	Radio button	Descriptive	Individual	0
			Assistance	0.5
			Dependent	1
Cleaning	Radio button	Descriptive	Individual	0
			Assistance	0.5
			Dependent	1
Shopping	Radio button	Descriptive	Individual	0
			Assistance	0.5
			Dependent	1
Meds	Radio button	Descriptive	Individual	0
			Assistance	0.5
			Dependent	1
Driving	Radio button	Descriptive	Individual	0
			Assistance	0.5
			Dependent	1
Banking	Radio button	Descriptive	Individual	0
			Assistance	0.5
			Dependent	1
Enough income?	Radio button	Descriptive	Yes	0
			No	1
Socially Engaged	Radio button	Descriptive	Frequent	0
			Occasional	0.5
			Not	1
Lives	Radio button	Descriptive	Spouse	0
			Other	0.5
			Alone	1
Number of Problems	Dropdown list	Numerical [0, 99]	Any integer between 0 and 18	Count of the total number of problems if ≤18; or 18 if it is > 18.
Number of Medications	Dropdown list	Numerical [0, 99]	≤4	0
			5-7	0.5
			≥8	1

Note: *The default value for all dropdown lists is "Select a value", there is no default value for the rest of the field types, MOCA = Montreal Cognitive Assessment, FAST = Functional Assessment Staging Test, FTST = Five Times Sit to Stand.*
