# Supplementary figures and images for: A Computerized Frailty Assessment Tool at Points-of-Care: Development of a Standalone Electronic Comprehensive Geriatric Assessment/Frailty Index (eFI-CGA)
Source: Front Public Health. 2020 Mar 31;8:89. doi: 10.3389/fpubh.2020.00089 (PMC7137764; doi:10.3389/fpubh.2020.00089)

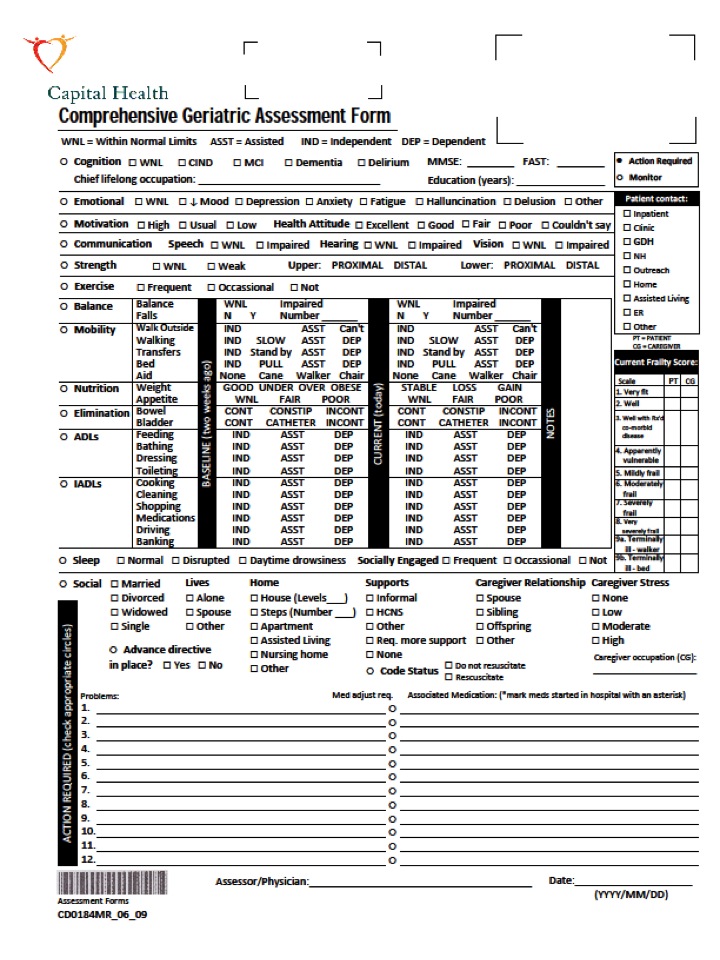

Supplement: Supplementary file 3 [file Image_1.jpg]
